# Disruption of Hyaluronic Acid in Skeletal Muscle Induces Decreased Voluntary Activity via Chemosensitive Muscle Afferent Sensitization in Male Mice

**DOI:** 10.1523/ENEURO.0522-21.2022

**Published:** 2022-04-13

**Authors:** Luis F. Queme, Adam J. Dourson, Megan C. Hofmann, Ally Butterfield, Rudolph D. Paladini, Michael P. Jankowski

**Affiliations:** 1 Department of Anesthesia, Division of Pain Management, Cincinnati Children’s Hospital Medical Center, Cincinnati, OH 45229; 2 Department of Anesthesiology, University of Cincinnati, Cincinnati, OH 45229; 3 Halozyme, Inc, San Diego, CA 92121; 4 Department of Pediatrics, University of Cincinnati College of Medicine, Cincinnati, OH 45229; 5 Pediatric Pain Research Center, Cincinnati Children’s Hospital Medical Center, Cincinnati, OH 45229

**Keywords:** behavior, dorsal root ganglia, electrophysiology, hyaluronidase, macrophage, voluntary activity

## Abstract

PEGPH20, a human recombinant hyaluronidase, has been proposed as a coadjutant to pancreatic cancer chemotherapy. In early trials, patients reported increased widespread muscle pain as the main adverse reaction to PEGPH20. To understand how PEGPH20 caused musculoskeletal pain, we systemically administered PEGPH20 to male mice and measured voluntary wheel activity and pain-related behaviors. These were paired with *ex vivo* electrophysiology of primary sensory neurons, whole DRG real-time PCR, and immunohistochemistry of hindpaw muscle. PEGPH20 induced significantly lower wheel running, compared with vehicle-treated animals, and decreased mechanical withdrawal thresholds 5 d after PEGPH20 injections. Chemo-sensory muscle afferents showed increased responses to noxious chemical stimulation of their receptive fields (RFs) in the PEGPH20-treated group. This was correlated with upregulation of the NGF receptor TrkA, the transient receptor potential vanilloid type 1 (TRPV1) channel and ATP-sensitive channel P2X3 in the DRG. Immunohistochemistry of hindpaw muscles revealed damage to the muscle architecture and extensive infiltration of the tissue by cells of the myelomonocytic lineage 3 d after PEGPH20 injection. Peripheral macrophage ablation in macrophage Fas-induced apoptosis (MaFIA) mice, however, did not prevent the decreased voluntary activity and instead caused even lower levels of running. These results suggest that disruption of hyaluronic acid (HA) within the muscle extracellular matrix (ECM) sensitizes chemo-nociceptive muscle afferents possibly leading to altered pain-like behaviors. Ablation experiments suggest macrophages are necessary for adequate recovery of voluntary activity after HA disruption. These data support a role for HA and macrophages in tissue integrity and muscle pain development in patients taking PEGPH20.

## Significance Statement

Hyaluronidase co-administration has been suggested as a possible solution to improve the delivery of chemotherapeutic agents into difficult to access tissues. In clinical trials, patients receiving a systemic dose of hyaluronidase reported widespread pain as a side effect. Delivering hyaluronidase to mice, we found that they experienced decreased voluntary activity. We observed alterations in the response properties of metabo-nociceptive muscle afferents, accompanied by increased muscle infiltration of myeloid lineage cells including macrophages. Macrophage depletion at the time of hyaluronidase administration surprisingly exacerbated the decrease in voluntary activity. This suggests that increased hyaluronidase levels can affect muscle function and lead to immune responses but also suggest these cells may be needed for muscle recovery to allow animals to perform activity-based tasks.

## Introduction

Pain is a frequent, undesired effect of pharmacotherapies aimed at treating different conditions. In the case of therapeutics used for cancer, long-lasting pain is a major problem. Recent studies have explored the use of a novel approaches to cancer therapeutics, such as the disruption of the tumoral stroma. One of the suggested approaches to achieve disruption of the tumor microenvironment includes the use of a pegylated recombinant human hyaluronidase (PEGPH20) to target hyaluronic acid (HA), a major component of the tumoral extracellular matrix (ECM). Initial, phase Ib trials exploring the use of this strategy in patients with pancreatic cancer, however, reported musculoskeletal pain as well as extremity pain accompanied by edema and fatigue as the most common PEGPH20-related adverse events (Hingorani et al., 2016). At sufficient doses (1 mg/kg), PEGPH20 increased the entry of therapeutic antibodies into the tumor stroma (Manuel et al., 2015; Singha et al., 2015; Li et al., 2018). Thus, it is important to characterize the mechanism behind the observed side effects to maximize the potential benefits of this approach.

HA is an important component of the ECM within the skeletal muscles. It has been localized to the epimysium, perimysium, and endomysium (Laurent et al., 1991; Piehl-Aulin et al., 1991). It has also been associated with the perivascular and perineural connective tissue (Laurent et al., 1991; Piehl-Aulin et al., 1991). In contrast, smooth muscle seems almost devoid of HA (Laurent et al., 1991). As such, HA plays an important role in maintaining the healthy architecture of skeletal muscle. A previous report has shown that HA can be pro-nociceptive or anti-nociceptive when injected in the skin, depending on its molecular weight (Ferrari et al., 2016). Low molecular weight HA, a subproduct of hyaluronidase activation and subsequent degradation of ECM HA during injury and inflammation, seems to be pronociceptive (Ferrari et al., 2016, 2018). Contrary to this, high molecular weight HA can be antinociceptive and ameliorate inflammatory pain (Ferrari et al., 2018; Bonet et al., 2020). Regardless of their opposing functions, both forms seem to be related to activation of the CD44 receptor in sensory neurons.

In contrast with the adverse effects reported in cancer patients, hyaluronidase has been used as a therapeutic agent to treat upper limb muscle stiffness in individuals with cerebral injury (Raghavan et al., 2016), without clinically significant side effects. The potential therapeutic benefit has also been reported in patients with myofascial pain syndrome (Ghasemi et al., 2020). These reports showing results in opposition to the adverse effects observed in larger clinical trials in cancer patients, highlight the need to better understand the effects of systemic administration of hyaluronidases in the skeletal muscles and the mechanisms behind its effects on musculoskeletal pain.

In order to develop strategies to ameliorate the pain-related adverse effects of the administration of hyaluronidase, we explored the effects of systemic PEGPH20 administration in mice. Our working hypothesis was that intraperitoneal administration of PEGPH20 will induce widespread muscle pain potentially because of disruption of the normal muscle architecture that affects the sensory processing. To test this, we used a wheel running activity assay, along with evoked withdrawal to muscle squeezing, and grip strength, known methods to evaluate painful responses from the muscles in mice (Tappe-Theodor et al., 2019; Contreras et al., 2021) to examine the effects of PEGPH20 administration. We verified the effects of PEGPH20 on muscle architecture via immunohistochemical staining of the hindpaw muscles and evaluated potential changes in afferent function using a muscle-nerve-dorsal root ganglia-spinal cord (SC) *ex vivo* preparation and real-time PCR on affected DRGs.

## Materials and Methods

### Animals

Experiments were conducted with young adult male mice (three to eight weeks) of the following backgrounds: C57BL/6J (C57), B6.129P2-Lyz2^tm1(cre)Ifo^/J (LysMcre), B6.Cg-Gt(ROSA)26Sor^tm14(CAG-tdTomato)Hze^/J (tdTom), C57BL/6-Tg(Csf1r-EGFP-NGFR/FKBP1A/TNFRSF6)2Bck/J also known as macrophage fas-induced apoptosis (MaFIA) mice (The Jackson Laboratory) and littermate controls. All animals were housed in a barrier facility in group cages of no more than four mice, maintained on a 12/12 h light/dark cycle with a temperature-controlled environment, and given food and water ad-libitum. All procedures were approved by the Cincinnati Children’s Hospital Medical Center Institutional Animal Care and Use Committee and adhered to National Institutes of Health Standards of Animal Care and Use under American Association for Accreditation of Laboratory Animal Care-approved practices.

### Administration of PEGPH20 and AP compound

A single dose of PEGPH20 or its vehicle solution (10 nm histidine, 130 nm NaCl, pH 6.5; provided by Halozyme) was administered at a dose of 1 mg/kg, intraperitoneally; the same effective dose used in clinical trials (Li et al., 2018). The designer drug AP20187 (AP) was administered to the MaFIA mice intraperitoneally for 7 d at a dose of 10 mg/kg for experiments using these animals.

### Evoked pain-related behaviors

Testing of pain-related behaviors, withdrawal thresholds to muscle squeezing and grip strength, have been performed previously (Ross et al., 2014; Queme et al., 2016, 2020). Mice were first tested at baseline, as well as 1, 3, 5, and 7 d after the administration of either PEGPH20 or vehicle, which were administered immediately after the baseline measurements.

Mechanical withdrawal thresholds were determined by squeezing the hindpaw muscle with a modified Paw Pressure Meter (World Precision Instruments), fitted with a rounded blunt probe that applied pressure to the plantar surface to stimulate deeper tissues. The withdrawal threshold was recorded for three trials with 5-min intervals between stimulations, and the average was used for analysis. Muscle strength was tested via a grip strength meter (BioSeb). Animals were held by the tail over a metal bar while support was provided for the forepaws to measure grip strength exclusively on the hindpaw. Once the animal firmly held the bar with its hind paws, they were quickly pulled back horizontally (along the axis of the force sensor) until they could not retain their grip. Grip strength was measured (in grams) in three rounds of three trials each, with 5 min between each round. The average of the nine trials was used for analysis.

### Wheel running protocol

Animals were transferred from home cages to new cages fixed with a voluntary wheel attachment (Lafayette Instruments). Animals were singly housed with free access to food and water. The animals used in these experiments were used solely for the acquisition of voluntary wheel running behaviors and were not used for any measurements of pain-like behaviors or tissue analyses as previous literature (Grace et al., 2016; Ross et al., 2018) suggests a large role for voluntary wheel running on pain-like responses. For that same reason, voluntary activity was only monitored immediately after the administration of the PEGPH20 or vehicle, in an attempt to get a clear picture of the effects of the compound without the confounder of previous exercise. Either C57Bl/6 wild-type (WT) or MaFIA animals were used for these experiments. In groups treated with AP, seven daily intraperitoneal injections (10 mg/kg) were administered 3 d before entry into the wheel cage and up to 4 d following entry. For all animals, PEGPH20 or vehicle was injected immediately before transfer to the wheel cages. Animals were housed in the wheel cages for 7 d. Measurements obtained from voluntary running included numbers of revolutions, mean velocity (m/min), and distance traveled obtained in hourly bins.

### *Ex vivo* recording preparation

*Ex vivo* recording was performed as previously described (Dourson et al., 2021). Briefly, mice were anesthetized with an intramuscular hindlimb injection of ketamine and xylazine (90 and 10 mg/kg, respectively) into the left leg and perfused transcardially with ice-chilled, oxygenated (95% O_2_-5% CO_2_) artificial CSF (aCSF; in mm: 127.0 NaCl, 1.9 KCl, 1.2 KH_2_PO_4_, 1.3 MgSO_4_, 2.4 CaCl_2_, 26.0 NaHCO_3_, and 10.0 D-glucose). The right hindpaw and the SC were then excised and placed in a bath of the same aCSF. The skin was removed along with the cutaneous branches of all the nerves. The SC was hemisected and the tibial nerve along with the distal hindlimb muscles innervated by this nerve (with bone left intact), were dissected in continuity with their respective DRGs (L5, L4, and L3). After dissection, the preparation was transferred to a separate recording chamber containing cold oxygenated aCSF. The paw was pinned on an elevated platform, keeping the entire paw perfused in a chamber isolated from the DRGs and the SC. Finally, the bath was slowly warmed to 32°C before recording from the DRGs.

All single-unit recordings were made from the L3 and L4 DRGs. Sensory neuron somata were impaled with quartz microelectrodes (impedance > 150 MΩ) containing 5% Neurobiotin (Vector Laboratories) in 1 m potassium acetate. Electrical search stimuli were delivered through a suction electrode on the tibial nerve to locate sensory neurons with axons in this nerve. The latency from the onset of this stimulus and the conduction distance between the DRG and the stimulation site (measured directly along the nerve), were used to calculate the conduction velocity (CV) of the fibers. Group IV afferents were classified as those with a CV ≤ 1.2 m/s, and Group III afferents were those with CVs between 1.2 and 15 m/s. Peripheral receptive fields (RFs) in the muscles were localized by electrically stimulating the muscles with a concentric bipolar electrode. Only driven cells with RFs in the muscles then underwent mechanical, thermal, and chemical testing. Mechanical response characteristics were assessed with an increasing series of von Frey hairs ranging from 0.4 to 10 g (with diameters of 0.23–0.36 mm). Mechanical stimulation of the RF was held for ∼1–2 s. Thermal responses were determined by applying hot (≥50°C) or cold (≤3°C) saline directly to the paw muscles at the electrically determined RF. Each application lasted ∼1–2 s. After that, the muscles were exposed to an oxygenated “low” metabolite mixture (15 mm lactate, 1 μm ATP, pH 7.0) and then to a “high” metabolite mixture (50 mm lactate, 5 μm ATP, pH 6.6; Jankowski et al., 2013; Queme et al., 2020). delivered by a valve controller with an in-line heater to maintain solutions at bath temperature. ATP was added to the mixture immediately before delivery of metabolites. Adequate recovery times (∼20–30 s) were employed between stimulations. All elicited responses were recorded digitally for off-line analysis (Spike2 software, Cambridge Electronic Design). Because exposure to metabolites can alter the response properties of sensory neurons, all mechanical and thermal stimuli were repeated after exposure to metabolites. To verify that the quality of the recordings does not deteriorate though the duration of the experiment, responses from afferents recorded at the beginning and end of the experiment were grouped within conditions and compared. We did not detect significant differences between recorded units recorded at the beginning versus the end of the experiment.

### Immunohistochemistry

In order to evaluate the integrity of the ECM and skeletal muscle fibers, we injected mice with 200 μl of Evans-blue dye (intraperitoneal, 1% in 0.9% sterile saline solution) immediately before administration of PEGPH20. Evans-blue dye (EBD) has been used extensively to evaluate the integrity and permeability of the membrane of muscle fibers (Hamer et al., 2002). Wheat germ agglutinin (WGA) conjugated with FITC (Life Technologies) was used to co-stain the tissue to visualize the membranes in the skeletal muscle, as previously described (Kostrominova, 2011). Briefly, muscle tissue was embedded in Tissue-Tek O.C.T. compound (Sakura Finetek USA Inc.), flash frozen in liquid nitrogen and sectioned at 10 μm on a cryostat and mounted on slides. Tissue was fixed on slide using 4% paraformaldehyde (PFA) in 0.1 m PBS. The samples were subsequently washed, blocked in 0.01 m PBS containing 5% horse serum, 1% bovine serum albumin, and 0.2% Triton X-100 for 10 min. Sections were stained with WGA-FITC (1:100), incubated for 1 h, washed and coverslipped. A separate set of muscle samples from mice expressing a florescent reporter (tdTomato) on cells of myelomonocytic lineage (macrophages) were co-stained with Dystrophin (rabbit anti-dystrophin 1:250; Abcam, catalog #ab15277), incubated overnight and labeled with secondary antibodies (Alexa Fluor 488, 1:400; Jackson immunoResearch) and coverslipped. Exposure time during microscopic analysis for each image was performed at the same intensity level to confirm staining above background. Distribution of fluorescent staining was determined with a Nikon confocal microscope with sequential scanning to avoid bleed-through of the fluorophores. Images were captured at 40× magnification. Three nonconsecutive sections, separated at least by four sections, from three different animals per condition were used to quantify the percentage of myofibers positive for EBD. All the muscle cells in a section were labeled using ImageJ and muscle cells that were observed to contain red staining were considered positive. The percentage of positive cells obtained from each animal was used for comparisons. Similar methods were used to quantify LysM;tdTom cells in the muscle. Macrophage ablation after AP compound administration was confirmed via confocal imaging of the full hindpaw of the mouse. As before, three nonconsecutive sections from three different animals per condition were used to quantify the area covered by GFP signal via ImageJ software. Minimum intensity threshold was set at 60 and maximum at 255. Average intensity per animal was determined among the three sections and data were then averaged across animals per group for comparisons. Images for publication were prepared using Photoshop Elements software (Adobe).

### RNA isolation and real-time PCR

DRG (L3–L4, right side) tissue was collected from PEGPH20, or vehicle-treated conditions at different time points. Tissue RNA was isolated using the Qiagen RNeasy kit, according to the manufacturer’s protocol. Fort real-time PCR, 500 ng of total RNA was DNase I treated (Invitrogen) and reverse transcribed using Superscript II (Invitrogen) reverse transcriptase. A total of 20 ng of cDNA were used in SYBR Green real-time PCRs that were performed in duplicate and analyzed on a Step-One real-time PCR machine (Applied Biosystems).

Primer sequences for GAPDH, ASIC3, transient receptor potential vanilloid type 1 (TRPV1), TRPA1, TrkA, GFRα3 were obtained from previously published work (Elitt et al., 2006). Primer sequences used for GFRα1, GFRα2, and P2X3 were obtained from work previously published (Jankowski et al., 2009). Primer sequences for IL1r1 and P2X5 have been reported previously (Ross et al., 2014). Cycle time (Ct) values for all targets were all normalized to a GAPDH internal control. Ct values (used to determine fold change after injury) were then obtained by subtracting the normalized target genes. Ct value from naive controls. Then fold change was determined as 2^ΔΔCt^ (Applied Biosystems). The error of the difference in means is then also calculated for the fold change. Values were then converted and reported as a percent change where 2-fold change = 100% change.

### Statistical analysis

All values are presented as mean ± SEM unless stated differently. Behavioral assays, RT-qPCR data, and comparisons of electrophysiological responses were tested with a one-way ANOVA, or a two-way repeated measures (RM) ANOVA with Bonferroni’s *post hoc* test when appropriate. Two group comparisons that failed normality tests were tested with a Mann–Whitney *U* test. Percentage of EBD positive cells was compared using a χ^2^ test. Critical significance level was set at *p* < 0.05 (Table 1).

**Table 1:** Statistics table

	Data structure	Type of test	Comparison	95% confidence interval
a	Normally distributed	Two-way RM ANOVA		
		Bonferroni *post hoc* test	PEGPH20 vs API buffer day 1	368.4–7286
		Bonferroni *post hoc* test	PEGPH20 vs API buffer day 2	67.36–6985
		Bonferroni *post hoc* test	PEGPH20 vs API buffer day 3	695.6–7613
b	Normally distributed	Two-way RM ANOVA		
		Bonferroni *post hoc* test	PEGPH20 vs vehicle day 5	11.85–146.1
c	Normally distributed	Two-way RM ANOVA	Bonferroni *post hoc* test	−8.005–12.52
d	Non-normally distributed	Kruskal–Wallis test		Test does not generate confidence interval
e	Non-normally distributed	Mann–Whitney *U* test		−1.000–3.000
f	Non-normally distributed	Mann–Whitney *U* test		−1.000–2.000
g	Non-normally distributed	Mann–Whitney *U* test		−80.66–89.10
h	Non-normally distributed	Mann–Whitney *U* test		1.700–24.62
i	Non-normally distributed	Mann–Whitney *U* test		−12.70–24.30
j	Non-normally distributed	Mann–Whitney *U* test		1.700–24.62
k	Normally distributed	χ^2^ test		Test does not generate confidence interval
l	Normally distributed	Unpaired *t* test		22,155–108,900
m	Normally distributed	Mixed-effects analysis	PEGPH20 vs vehicle day 1	−7169 to −963.0
		Bonferroni *post hoc* test	PEGPH20 vs vehicle day 2	−6877 to −456.6
		Bonferroni *post hoc* test	PEGPH20 vs vehicle day 3	−8772 to −279.7
		Bonferroni *post hoc* test	PEGPH20 vs PEGPH20+AP day 4	110.9–2674
		Bonferroni *post hoc* test	PEGPH20 vs PEGPH20+AP day 5	309.0–2507
		Bonferroni *post hoc* test	PEGPH20 vs PEGPH20+AP day 6	389.1–3866
		Bonferroni *post hoc* test	PEGPH20 vs PEGPH20+AP day 7	580.0–3549
n	Normally distributed	One-way RM ANOVA		
		Bonferroni *post hoc* test	PEGPH20 vs PEGPH20+AP	395.7–2221
		Bonferroni *post hoc* test	PEGPH20 vs vehicle	−4459 to −2177
		Bonferroni *post hoc* test	PEGPH20+AP vs vehicle	−5256 to −3997
		Bonferroni *post hoc* test	AP vs vehicle	1464–4666

## Results

### PEGPH20 induces long-lasting reductions in voluntary activity as well as increased pain-related behaviors

**Figure 1.**
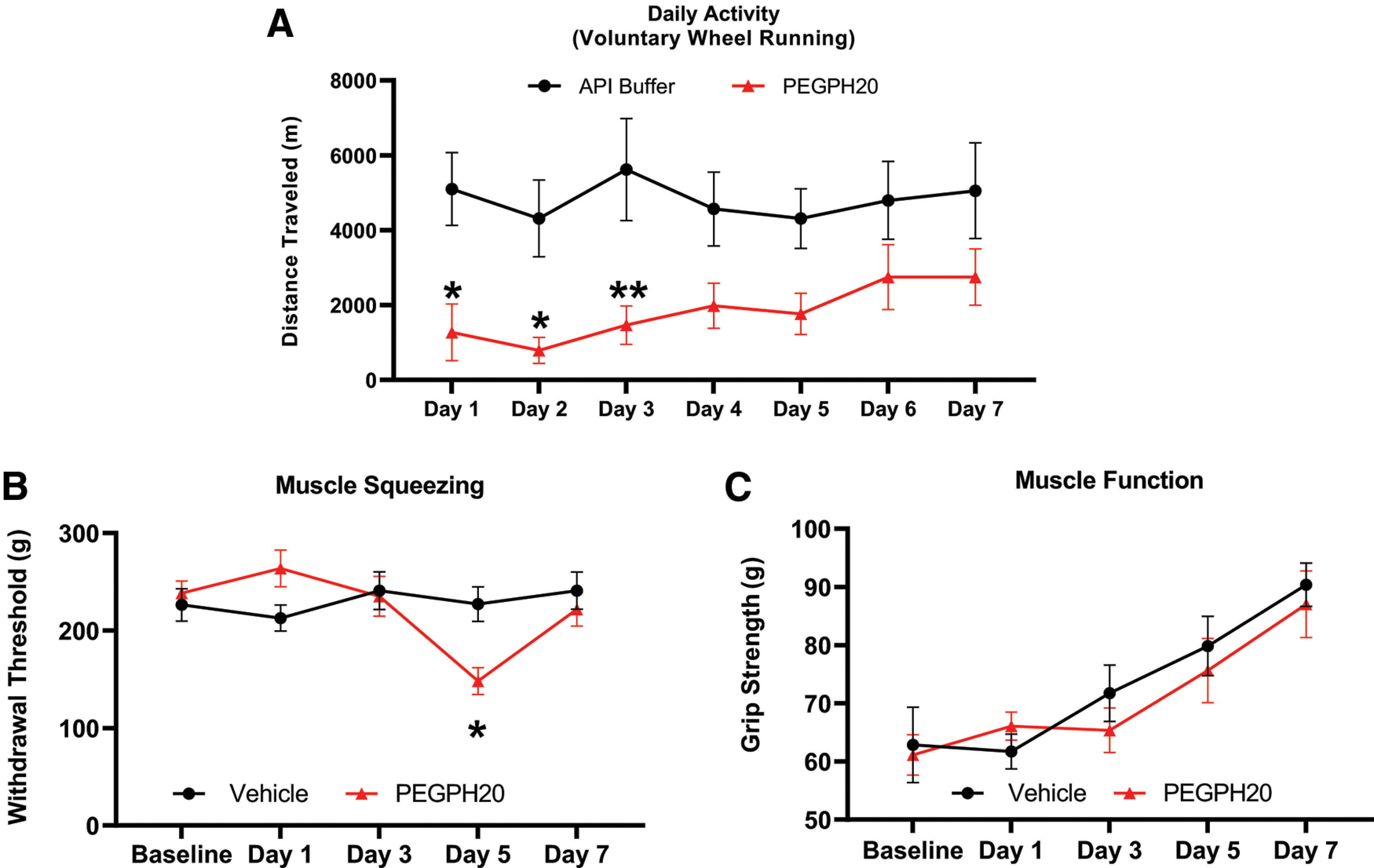
PEGPH20 decreases voluntary wheel running but does not cause acute mechanical hypersensitivity. ***A***, Voluntary wheel running is significantly decreased up to 3 d after the administration of PEGPH20 (*n* = 12), compared with vehicle controls (*n* = 12). ***B***, Withdrawal thresholds to muscle squeezing are significantly lower 5 d after the administration of PEGPH20 (*n* = 8) compared with vehicle-treated mice (*n* = 8). ***C***, There are no significant differences in grip strength between mice treated with PEGPH20 (*n* = 8) versus vehicle-treated mice (*n* = 8). Two-way RM ANOVA (Bonferroni *post hoc*); ***A***, *F*_(1,14)_ = 6.654, *p* = 0.218; ***B***, *F*_(4,56)_ = 5.515, *p* = 0.0008; ***C***, *F*_(4,56)_ = 0.68. ***A–C***, *p < 0.05. **p <0.01 versus vehicle.

Based on previous reports, we evaluated the effect of PEGPH20 on the activity levels of uninjured mice. To do this, we administered PEGPH20 or vehicle solution at 1 mg/kg intraperitoneally. The mice injected with vehicle ran approximately the same distance every day, preferentially during the night phase of the illumination cycle. Immediately after the administration of PEGPH20, the mice showed significantly lower activity than their vehicle-injected counterparts (Fig. 1*A*^a^). Activity remained significantly lower during the first 3 d after the administration of PEGPH20 and did not recover to the same level of activity observed in vehicle-treated animals during the testing period.

In separate cohorts of mice, we tested pain related behaviors 1 d before the administration of PEGPH20 or vehicle and continued testing 1 d after the administration and every other day up to 7 d (Fig. 1*B*^b^). We did not detect any changes in the mechanical withdrawal thresholds to muscle squeezing at 1, and 3 d after compound administration. In contrast, 5 d after the administration of PEGPH20 there was a significant decrease in the mechanical withdrawal threshold. This decrease in withdrawal threshold reversed 7 d after PEGPH20 administration. Finally, we tested grip strength in the hind paws. While the mean grip strength increased slowly after day 1 (Fig. 1*C*^c^), both groups followed the same pattern and there were not significant differences between the group that received PEGPH20 or the group that received vehicle.

### PEGPH20 induces changes in the response properties of primary sensory neurons to a noxious combination of metabolites

**Figure 2.**
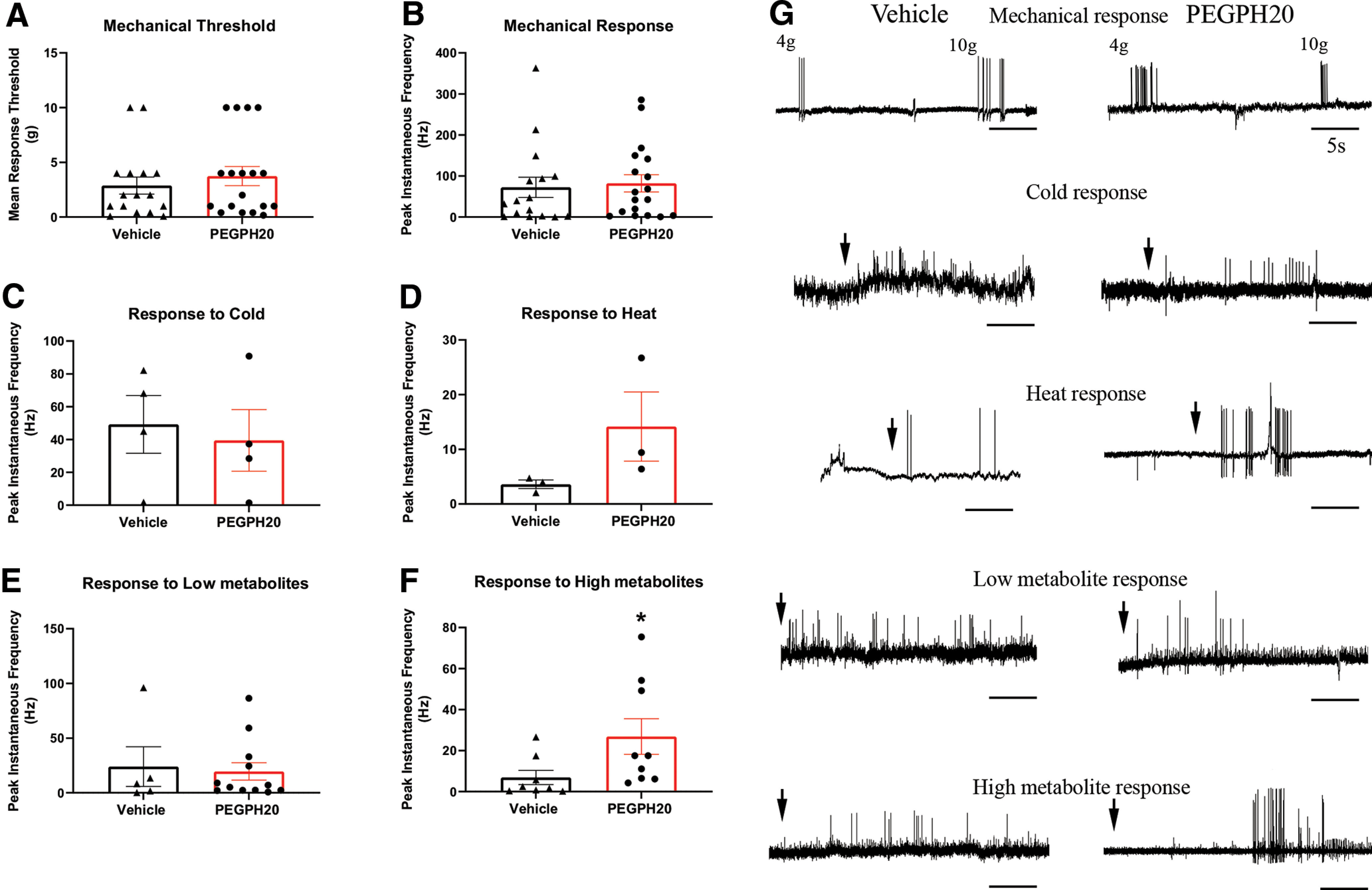
PEGPH20 induces increased response to noxious metabolites in metabo-nociceptive primary muscle afferents. ***A–D***, We did not observe changes in the mechanical thresholds or response patterns of mechanically sensitive neurons after PEGPH20 (*n* = 18) administration, compared with vehicle controls (*n* = 18). The same can be said of the responses to heat (*n* = 3 per group) or cold (*n* = 4 per group) stimulation. ***E–F***, While there were no changes in the response to chemical stimulation in neurons sensitive to a low concentration of metabolites (vehicle, *n* = 5; PEGPH20 *n* = 12), injection of PEGPH20 caused a significant increase in the instantaneous frequency of firing in high metabolite responsive neurons (metabo-nociceptors; vehicle *n* = 8, PEGPH20 *n* = 9). ***G***, Representative traces of mechanical, thermal, and chemical responses from neurons recorded from either vehicle or PEGPH20-treated mice. Arrows represent application of stimulus. ***A–F***, Mann–Whitney *U* test, *p < 0.02 versus vehicle.

Using an *ex vivo* muscle-nerve-DRG-SC preparation, we performed single-unit recordings from afferents innervating the right hindpaw muscles at 1 and 3 d after the administration of PEGPH20 or vehicle. Preliminary analysis did not reveal differences between the recordings captured at 1 or 3 d for both groups as shown in Table 2, so both timepoints were merged into one group for each condition. As shown in Figure 2*A*,*B*^e,f^, we did not find any significant differences in the afferent response properties to mechanical stimulation. No changes in either threshold, instantaneous frequency (IF), or firing rate (FR) were observed between conditions. There were also no significant differences in the responses to heat or cold stimulation (Fig. 2*C*,*D*^g,h^). However, when we stimulated the preparation with different concentrations of metabolites (lactate, ATP, and low pH) resembling the environment in the muscle during normal work-related activity (low metabolites) or ischemic contractions (high metabolites), we found no differences in the response pattern to the low concentration of metabolites, but found an increased response of Group III/IV afferents (IF) to higher concentrations of these metabolites in the group that was treated with PEGPH20 compared with vehicle-treated controls (Fig. 2*E*,*F*^i,j^).

**Table 2:** General electrophysiological parameters from recordings 1 and 3 d after administration of PEGPH20 or vehicle

	Condition	
	Vehicle 1 d	Vehicle 3 d
Conduction	10.56 ± 1.99	6.71 ± 1.37
velocity	(*n* = 27)	(*n* = 21)
Mechanical threshold	2.77 ± 1.08	4.29 ± 1.52
(*n* = 9)	(*n* = 7)	
Mechanical response	44.02 ± 18.57	108.80 ± 50.19
(peak IF, Hz)	(*n* = 9)	(*n* = 7)
Heat response	3.03 ± 0.95	4.7 ± 0.0
(peak IF, Hz)	(*n* = 2)	(*n* = 1)
Cold response	63.53 ± 18.53	34.90 ± 33.20
(peak IF, Hz)	(*n* = 2)	(*n* = 2)
Low metabolite response	13.4 ± 0.0	26.58 ± 23.24
(peak IF, Hz)	(*n* = 1)	(*n* = 4)
Hight metabolite response	1.39 ± 0.64	8.37 ± 6.19
(peak IF, Hz)	(*n* = 3)	(*n* = 4)
	Condition	
	PEGPH20 1d	PEGPH20 3d
Conduction	6.487 ± 1.18	10.33 ± 1.92
velocity	(*n* = 24)	(*n* = 24)
Mechanical threshold	5.2 ± 1.11	1.92 ± 1.17
(*n* = 9)	(*n* = 7)	
Mechanical response	77.41 ± 9.95	88.07 ± 31.53
(peak IF, Hz)	(*n* = 9)	(*n* = 7)
Heat response	26.7 ± 0.0	7.9 ± 1.5
(peak IF, Hz)	(*n* = 1)	(*n* = 2)
Cold response	No cold responses recorded	39.48 ± 18.73
(peak IF, Hz)	(*n* = 4)	
Low metabolite response	3.9 ± 1.45	30.66 ± 12.09
(peak IF, Hz)	(*n* = 5)	(*n* = 7)
Hight metabolite response	13.16 ± 4.62	37.82 ± 14.05
(peak IF, Hz)	(*n* = 4)	(*n* = 5)

We do not observe any significant differences between electrophysiological recordings performed either 1 or 3 d after treatment in each condition.

### PEGPH20 causes a breakdown of the membranes within the muscle as well as an increased infiltration by LysM-tdTom cells

**Figure 3.**
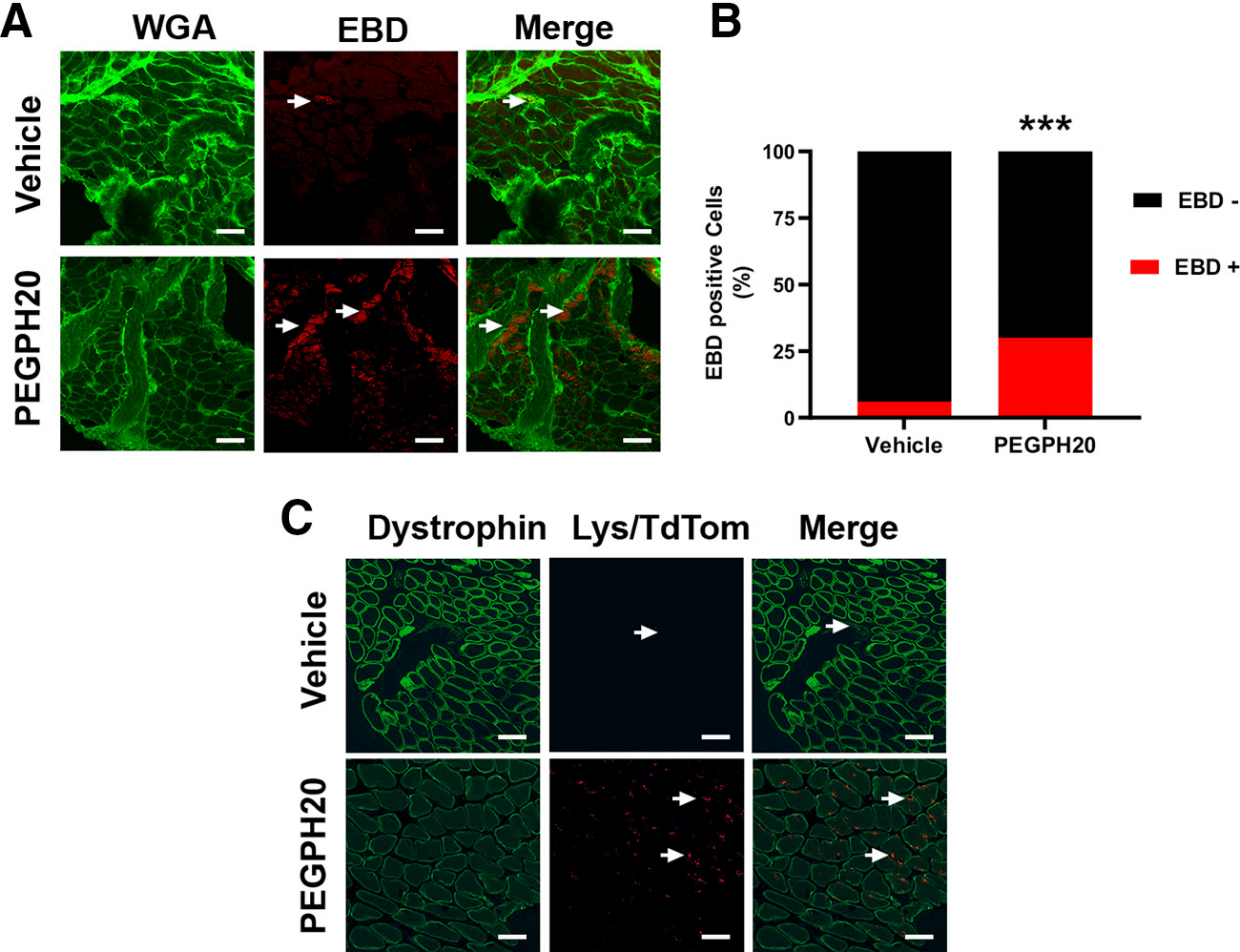
PEGPH20 disrupts of skeletal muscle architecture that is accompanied by macrophage infiltration. ***A***, Muscle extracellular membrane (green, WGA) is disrupted 3 d after the administration of PEGPH20. EBD (red) usually remains in intravascular spaces in intact tissue but after hyaluronidase administration it leaks into the affected skeletal muscle. ***B***, Percentage of myofibers positive for EBD 3 d after PEGPH20 administration. ***C***, At the same time point macrophages (red, LysM/tdTomato) infiltrate the connective tissue surrounding the skeletal muscle in contrast to the almost nonpresent macrophages in the vehicle-treated animals. White scale bar: 50 μm. ***p < 0.001 χ^2^ test.

The target of PEGPH20 is HA, an important structural component of the architecture of skeletal muscle. We suspected that the effects of PEGPH20 on voluntary activity and response properties of skeletal muscle afferents could be explained by changes in the structure of the muscle. As shown in Figure 3*A*,*B*^k^, 1 d after the administration of PEGPH20, we observed widespread staining of the muscle tissue with Evans-blue dye, suggesting a disruption of the membranes within the skeletal muscles. In the mice treated with vehicle, there is very low Evans-blue leakage into the muscle.

Tissue injury has frequently been associated with increased infiltration of the affected tissue by immune cells. In particular, macrophages are known to play an important role not only in the development of muscle pain in other models but have also been suggested to play an important role in the subsequent repair of the injured tissue. In order to evaluate whether macrophages could be playing a role in the increased musculoskeletal pain secondary to PEGPH20 administration, we administered PEGPH20 to mice expressing a fluorescent reporter protein in cells of myelomonocytic lineage (LysMcre/TdTomato mice). Three days after the administration of PEGPH20, we observed widespread infiltration of myeloid lineage cells into skeletal muscle (Fig. 3*C*) compared with little infiltration in the vehicle-treated animals (Veh: 3.9 ± 1.9 vs PEGPH20: *32 ± 3.4; *t* = 6.46, df = 4; *p < 0.003, *t* test).

To better understand the effects of the alterations in the musculoskeletal structure in the changes observed in response properties of primary muscle afferents, we isolated RNA from whole DRGs (L3 and L4) and performed real-time PCR. We tested for a variety of genes at 1 d after PEGPH20 administration. We did not observe significant changes in gene expression 1 d after PEGPH20 administration. Three days after injection of PEGPH20, however, we observed a significant upregulation in the P2X receptor 3 (P2X3), and a decrease in the expression of the GDNF receptor, GFRα1 and the Interleukin-1 receptor IL-1r1. By day 5, we observed a significant decrease in the expression of the NGF receptor TrkA reverting the trend observed in day 1 and day 3. No changes were observed at 7 d with the exception of P2X3 which remained elevated 7 d after injury (Table 3).

**Table 3:** Select DRG gene expression after PEGPH20 administration (1–7 d)

Gene	D1	D3	D5	D7
TrkA	21.9 ± 13.5	70.2 ± 8.0	−60.7 ± 13.4*	−52.2 ± 30.3
P2X3	49.8 ± 13.1	458.3 ± 13.8***	114.8 ± 29.2	459.6 ± 25.9**
P2X5	−4.4 ± 10.2	−63.9 ± 39.1	−37.0 ± 38.9	−62.8 ± 15.9
TRPV1	9.3 ± 15.2	58.6 ± 9.0	−66.3 ± 20.1	−63.4 ± 49.4
TRPA1	−23.8 ± 12.6	−13.66 ± 113.03	58.3 ± 28.6	−32.99 ± 37.53
ASIC3	0.8 ± 7.2	7.5 ± 5.0	−82.77 ± 110.1	−76.33 ± 61.62
GFRα1	−2.8 ± 7.3	−81.8 ± 12.1**	−93.1 ± 26.8***	−97.6 ± 56.1***
GFRα2	30.3 ± 18.8	−44.8 ± 95.9	−55.9 ± 41.4	−69.2 ± 29.4
GFRα3	−7.6 ± 5.5	−60.5 ± 120.6	−18.5 ± 37.31	−37.8 ± 14.72
IL1r1	−17.9 ± 11.9	−90.1 ± 85.3*	−83.3 ± 28.5	−89.1 ± 7.9*

Values indicate % change versus vehicle-treated controls. Mean ± SEM *n* = 4 per group per time point. One-way ANOVA (TrkA *F*_(4,14)_ = 10.57, *p* = 0.0004; P2X3 *F*_(4,14)_ = 17.27, *p* < 0.0001; GFRα1 *F*_(4,13)_ = 29.57, *p* < 0.0001; LI1r1 *F*_(4,12)_ = 8.272, *p* = 0.0019) with Bonferroni *post hoc*. *p < 0.05, **p < 0.01, ***p < 0.001 versus vehicle-treated controls. GAPDH CT (mean ± SEM) CTRL: 20.97 ± 0.26; D1 19.651 ± 0.07; D3 20.295 ± 0.45 D5 21.2 ± 0.39 D7 22.32 ± 0.82. No significant differences were detected between the CTRL or treatment groups (one-way ANOVA *F*_(4,14)_ = 3.8, *p* = 0.026).

### Ablation of macrophages does not improve the effects of PEGPH20 on voluntary activity

**Figure 4.**
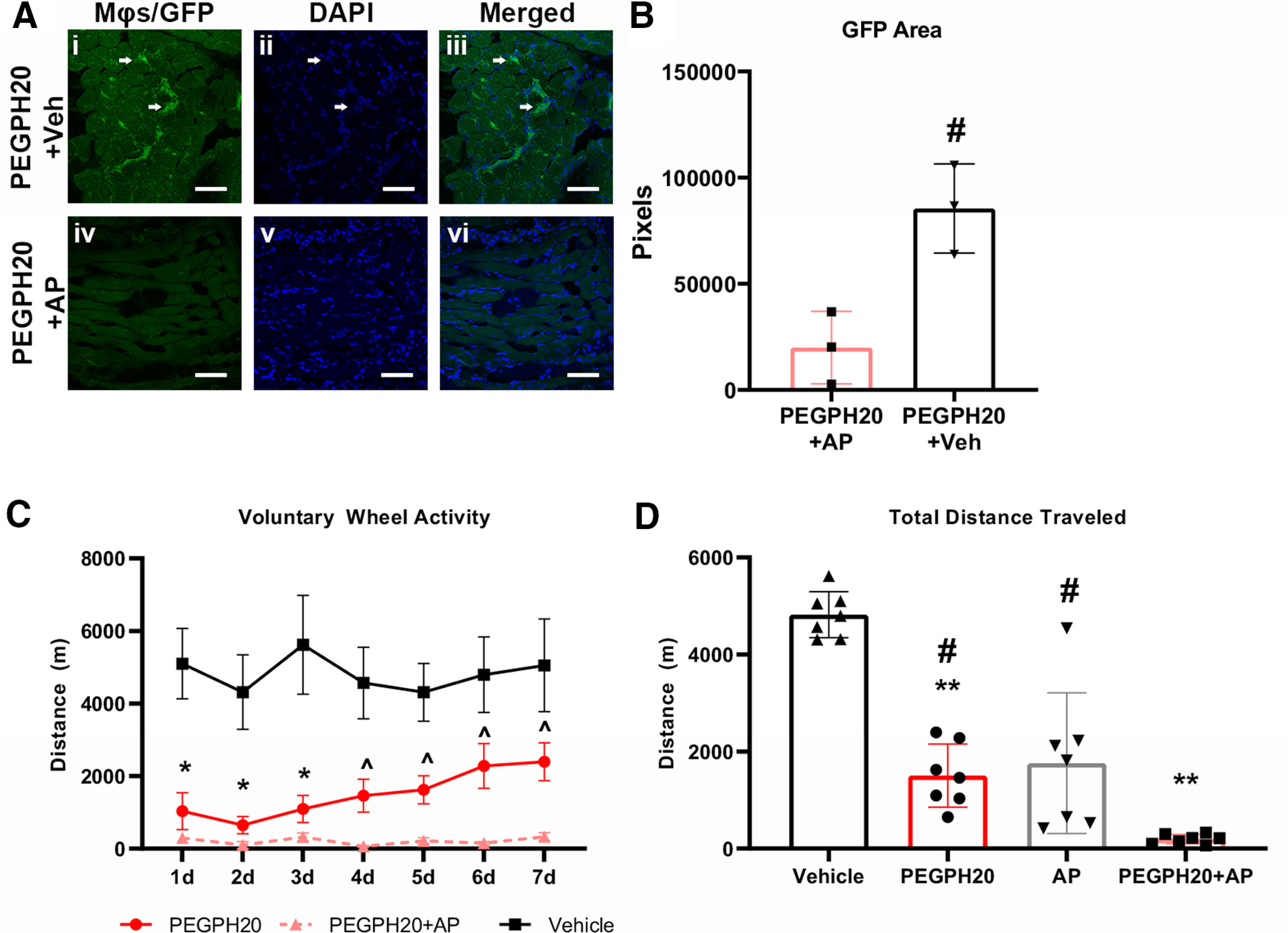
Ablation of macrophages does not prevent decreased voluntary wheel running after administration of PEGPH20. ***A***, Administration of AP for 7 d starting 3 d before PEGPH20 injection removes macrophages from all hindpaw tissues as revealed by the lack of GFP signal (arrows) in the treated animals compared with vehicle-treated controls. ***B***, Quantification of GFP signal reveals significantly less signal in AP-treated mice (*n* = 3) versus vehicle-treated animals (*n* = 3). ***C***, Ablation of macrophages in MaFIA mice does not prevent the development of decreased voluntary wheel running distance after PEGPH20 (*n* = 12) administration compared with vehicle-treated mice (*n* = 12). In fact, the combination of PEGPH20+AP (*n* = 8) used to ablate macrophages prevents the slow recuperation that initiates around 4 d after PEGPH20 administration. ***D***, Total wheel running distance is significantly lower in PEPH20, AP alone (*n* = 8), or PEGPH20+AP-treated mice compared with just vehicle-treated mice. The total running distance of PEGPH20+AP-treated mice was also significantly lower than in animals treated with just PEGPH20 or AP alone. White scale bar: 50 μm. ***B***, Unpaired *t* test; ***C***, Mixed-effects analysis (*F*_(2,29)_ = 19.16, *p* < 0.0001) with Bonferroni *post hoc*; ***D***, One-way ANOVA (*F*_(3,24)_ = 38.87, *p* < 0.001) with Bonferroni *post hoc*. #*p* < 0.05, ^*p* < 0.01 versus PEGPH20+AP; *p < 0.05, **p < 0.01 versus vehicle.

Our previous observation of infiltration by cells of myelomonocytic lineage in the skeletal muscle after PEGPH20 administration suggested that these immune cells may be playing a significant role in the development of long-lasting muscle pain. To test whether macrophages played any role in the development of decreased voluntary activity after PEGPH20 administration, we decided to ablate them in mice with PEGPH20 injection and assayed voluntary running. To do this, we used MaFIA mice. MaFIA mice are a transgenic line that, when injected with the designer drug AP temporarily ablates peripheral macrophages and dendritic cells. Our injection protocol replicated the dose and scheme used in previous studies that showed evident macrophage depletion 24 h after the third injection (Burnett et al., 2004). Other works using lower doses than our protocol report up to 90% depletion of circulating monocytes that does not recuperate until 4 d after the last dose of AP (Yu et al., 2020). We confirmed the effectiveness of this strategy via immunohistochemistry. MaFIA mice treated with PEGPH20 + AP compound had significantly less GFP signal in the hindpaw compared with PEGPH20 + vehicle-treated animals (Fig. 4*A*,*B*^l^). This approach insured that macrophages were not present in the periphery at the time of PEGPH20 administration and that the macrophage population would not start to recover until after at least 5 d into the running experiment, allowing for the observation of the voluntary activity pattern of the mice when exposed to hyaluronidase, but lacking peripheral macrophage infiltration.

We found that the injection of PEGPH20 reduced the distance that animals traveled in all groups compared with vehicle controls and that ablation of macrophages by injection of AP did not ameliorate the effects of PEGPH20. In fact, while animals injected with only PEGPH20 showed a small recovery of the traveled distance at days 6 and 7, the animals that received AP in combination with PEGPH20 did not show any recovery (Fig. 4*C*^m^). Administration of AP alone induced a significant decrease in activity compared with the vehicle-treated mice and almost in line with the decrease observed after administration of PEGPH20 (Table 4). This suggests that macrophage depletion can have a big impact on mouse behavior and should always be considered when using MaFIA mice as a model. When we compared the total running distance of mice injected with PEGPH20 or AP alone to the combination of both PEGPH20 and AP, the combination group showed an additive effect, reducing voluntary activity almost completely. We found that animals injected with both compounds ran significantly less than those injected with either one compound alone, as well as from controls (Fig. 4*C*^m^,*D*^n^). This suggests that the presence of macrophages in the skeletal muscle after the disruption of the architecture by hyaluronidase administration may be protective and may be more important for the recuperation of the mice from hyaluronidase treatment than for the development of the decreased voluntary activity.

**Table 4:** Total daily distance traveled after treatment

	Vehicle	PEGPH20	PEGPH20+AP	AP
1d	5100.08 ± 972.65 m	1034.07 ± 507.76 m*	297.27 ± 63.98 m**	2118.52 ± 622.81 m
2d	4314.84 ± 1028.88 m	647.95 ± 237.69 m*	97.19 ± 92.70 m*	529.68 ± 225.32 m*
3d	5617.62 ± 1360.53 m	1091.83 ± 372.54 m	319.24 ± 113.41 m*	4541.73 ± 2236.27 m
4d	4566.33 ± 987.13 m	1457.09 ± 454.28 m*	64.62 ± 22.11 m*	419.32 ± 340.40 m*
5d	4308.31 ± 793.60 m	1624.41 ± 386.50 m*	216.20 ± 85.72 m** ^#^	1819.74 ± 1368.44 m*
6d	4795.18 ± 1037.20 m	2278.82 ± 616.13 m	151.05 ± 40.59 m* ^#^	646.48 ± 496.81 m*
7d	5052.14 ± 1275.32 m	2393.98 ± 522.54 m	329.55 ± 108.71 m* ^#^	2223.02 ± 1497.26 m

Administration of AP alone (*n* = 6) induced changes similar to vehicle (*n* = 8), PEGPH20 (*n* = 12), and PEGPH20+AP (*n* = 12). the combination of PEGPH20+AP produced significantly lower levels of activity during the first 4 d after administration but did not show the recuperation observed by 7 d in both the PEGPH20 or AP alone treated groups. Data from AP alone for day 3 only includes *n* = 2 because of equipment failure that did not allow capture of data for the single time point. Two-way ANOVA, *F*_(3,34)_ = 12.54, *p* < 0.0001 with Bonferroni *post hoc* test. *p < 0.05, **p < 0.01 versus vehicle, #*p* < 0.05 versus PEGPH20.

## Discussion

Pharmacological therapies for cancer frequently result in the undesired adverse effect of pain (Fallon, 2013; Masocha, 2018). As the search for novel therapeutic approaches expands, it is logical to expect different clinical presentations. Recently, therapies involving the administration of hyaluronidase systemically as a potential enhancer of chemotherapeutic approaches for pancreatic cancer have been explored (Hingorani et al., 2016). One of the observed adverse effects of this approach was widespread muscle pain (Hingorani et al., 2016). In contrast, in other clinical settings, hyaluronidase has been used successfully in ophthalmic surgery as an enhancer of local anesthesia (Buhren et al., 2016; Rüschen et al., 2018). The potential of the enzyme to disrupt connective tissue adhesions has led to the off-label use of this enzyme for the treatment of epidural adhesions associated with chronic back pain (Dunn et al., 2010) or to treat myofascial pain syndrome secondary to tissue contractures (Raghavan et al., 2016; Ghasemi et al., 2020). Recent reports have explored the specific role of HA in the sensitization of primary sensory neurons. Several reports suggest that high-molecular-weight HA (HMWHA) can reduce inflammation-induced hyperalgesia (Ferrari et al., 2018; Bonet et al., 2020), while its counterpart, low-molecular-weight HA (LMWHA) is capable of inducing mechanical hyperalgesia (Ferrari et al., 2016). Furthermore, it has been reported that digestion of HMWHA by hyaluronidase produces LMWHA and that the later can inhibit the differentiation of monocytes that infiltrate tissue after injury into fibrocytes, cells that help in the repair of tissue after injury (Maharjan et al., 2011). These findings would be in line with our observations and would explain why the administration of PEGPH20 not only increases the recruitment of myeloid lineage cells such as macrophages into the affected muscle but would also explain why the removal of cells of myelomonocytic lineage aggravates the effects of PEGPH20 administration.

In this study, we aimed to further characterize the effects of systemic administration of PEGPH20 in the development of widespread pain and determine the contribution of muscle primary sensory neurons in this phenomenon. In accordance with clinical observations, our mice experienced an immediate decrease in voluntary wheel running after the administration of PEGPH20. Voluntary wheel running has been shown to be a reliable pain assessment tool in rodents (Ross et al., 2018; Kandasamy and Morgan, 2021) and has been shown to recuperate faster than evoked measurements, closely replicating the recovery observed in the clinical setting (Kandasamy et al., 2016). These differences between the results observed from voluntary running and the evoked pain-related behaviors (muscle squeezing), can explain why we are able to observe an acute drop in the activity, but we do not detect changes in withdrawal thresholds until 5 d after injection. It is also relevant that in our behavioral assessments, we did not observe a decrease in overall grip strength suggesting that the effects of PEGPH20 do not alter the ability to forcefully contract muscles and that the differences in activity are more representative of pain-like effects under these specific conditions.

The lack of changes in the mechanical withdrawal thresholds at 1 and 3 d after PEGPH20 administration correlates very well with the observations in our electrophysiological recordings where we did not detect any differences in the response to mechanical stimulation between groups. Nevertheless, a limitation of this study lies in the fact that we did not perform electrophysiological studies at 5 d after PEGPH20 administration that would facilitate the understanding of the evoked behavioral changes observed at this time point. However, it is interesting that we detected a significant difference in the responses to stimulation with high metabolites, a combination of low pH, ATP and lactic acid that closely resembles the environment in the muscle during painful, ischemic muscle contractions (Light et al., 2008; Jankowski et al., 2009; Pollak et al., 2014; Ross et al., 2014; Queme et al., 2020). If this specific subset of afferents, thought to perform the function of metabo-nociceptors is sensitized, it is logical to expect that during intense muscle activity, the mice would experience increased pain and thus avoid voluntary exercise. Since the changes in activity were so robust and were detected very early on, it is likely that sensitization of these afferents is a larger driving force of HA-induced pain in humans rather than changes in mechanical nociceptors. It is important to note that heat responsiveness in afferents is slightly, but not statistically increased after PEGPH20 treatment. A future study assessing greater numbers of heat sensitive afferent would be necessary however to determine this. Another important limitation of this study is that it was only performed in male mice. Several conditions presenting with chronic musculoskeletal pain, such as fibromyalgia, have a higher prevalence in females. It will therefore be important to perform these studies in females to assess sex differences in responses to PEGPH20.

Multiple receptors have frequently been associated with the development of musculoskeletal pain. Some of the receptors that have been traditionally associated with increased pain include the NGF receptor TrkA (Hayashi et al., 2011; Queme et al., 2013; Oga et al., 2020) as well as the ion channel TRPV1. Contrary to our expectations, neither of these receptors showed increased expression after the administration of PEGPH20. In fact, TrkA it was significantly downregulated 5 d after the administration of hyaluronidase. While these results are indicative of directionality, it is worth noting that further experimentation is needed to understand whether the mRNA expression profile observed after administration of PEGPH20 is also translated to protein levels (Schindler et al., 1990; Raqib et al., 1996; Donlin-Asp et al., 2021) Other receptors that have been frequently associated with the development of muscle pain in the context of ischemia, such as IL-1r1 (Ross et al., 2016), and the GDNF receptor GFRα1 (Queme et al., 2020) were also found to be downregulated at several timepoints after the administration of PEGPH20. Previous studies have shown that ischemic injury of the muscle can induce upregulation of these receptors. It is plausible that direct injury of the muscle cells is necessary to cause this upregulation and that disruption of the ECM is causing sensitization of primary muscle afferents via a completely different mechanism. In contrast, the P2X3 receptor is known to be upregulated in the DRG after ischemic muscle injuries (Ross et al., 2014, 2016) and has been linked to inflammatory pain development (Queme et al., 2017). This receptor may therefore play a role in the development of pain and decreased voluntary activity in these experiments. As an ATP sensitive ion channel, increased expression of P2X3 could be involved in the increased responses observed in the chemo-nociceptive muscle afferents.

The increased presence of myelomonocytic-lineage cells in the muscle tissue after PEGPH20 administration suggested an important role for these immune cells. We hypothesized that macrophage infiltration in the muscle may be playing a role in sensitizing primary muscle afferents as this has been shown in different models of musculoskeletal pain (Gong et al., 2016; Oliveira-Fusaro et al., 2020). We tested this by ablating the peripheral macrophage population during PEGPH20 administration. Contrary to our predictions, we did not observe a recuperation of the voluntary activity when we administered PEGPH20 in combination with AP. In fact, we observed a marked decrease in the total activity of the animals. Previous work has shown that after systemic depletion of macrophages, nerve injury related pain can be prevented (Yu et al., 2020). However, in that study, depletion of the macrophages infiltrating the DRG after nerve injury was required to prevent the development of pain. A recent study used a tourniquet-based model of complex regional pain syndrome (CRPS), to induce musculoskeletal pain and showed that depletion of macrophages could prevent the development of pain after ischemic injury (De Logu et al., 2020). These studies, show that macrophages are necessary for the development of musculoskeletal pain. In contrast with our observations, neither of the models used in the previous work, result in the disruption of the ECM and the potential generation of LMWHA that would be expected after the administration of hyaluronidase.

Other studies using the MaFIA mouse line have reported that AP compound administration can induce malaise, intra-abdominal tissue adhesion and weight loss in mice (Burnett et al., 2004; Yu et al., 2020). Interestingly, we found that AP alone induced a significant decrease in the overall activity levels comparable to the effect of PEGPH20 alone. A previous study using the MaFIA mouse line found that systemic administration of AP could cause significant weight loss and increased baseline mechanical thresholds (Yu et al., 2020). In the current study, we observed an additive effect in MaFIA animals treated with AP and PEGPH20, where animals had overall lower activity than either group alone. These data and the previous work indicate that the removal of myeloid-lineage cells results in both acute mechanical alterations, as well as prolonged prevention of physical activity. However, future studies using these animals should consider assessing multiple parameters as weight, mechanical thresholds, and activity are all affected by monocyte/macrophage ablation.

Macrophages are also known to play an important role in tissue repair after injury (Wynn and Vannella, 2016). They play an important role in the initiation maintenance and resolution phases of tissue repair (Mantovani et al., 2013; Wynn and Vannella, 2016; Kim and Nair, 2019). As such, it is possible that instead of preventing the development of muscle pain, macrophage ablation prevented the resolution of pain after PEGPH20 administration. This result is an important consideration for future therapies that desire to target macrophage depletion for pain relief.

Overall, this study highlights how systemic administration of hyaluronidase can disrupt the architecture of skeletal muscle, inducing sensitization of primary muscle afferents and leading to pain-like behaviors. Under these conditions, macrophages may be playing an important role in the resolution of injury instead of driving the initiation of pain. Our results also point out at the possibility that macrophages are needed to resolve damage to skeletal muscle and potential therapies should not focus exclusively in removing or inhibiting macrophages but consider the possibility of harnessing their injury resolving properties as potential therapeutic options.

## Synthesis

Reviewing Editor: Miriam Goodman, Stanford University Decisions are customarily a result of the Reviewing Editor and the peer reviewers coming together and discussing their recommendations until a consensus is reached. When revisions are invited, a fact-based synthesis statement explaining their decision and outlining what is needed to prepare a revision will be listed below. The following reviewer(s) agreed to reveal their identity: Theodore Price, Andrew Cooper.

In this submission, you investigate the effects of pegylated human recombinant hyaluronidase (PEGPH20) on muscle hypersensitivity and behavior using mouse models. The importance of the translational goal was appreciated by both reviewers, as was the quality of the research presented. That said, reviewers highlighted elements of the experimental design, figure presentation, and language precision that should be improved. In your revision, please address both of the reviewers’ specific comments (appended below).

Editorially, I wish to draw your attention to four especially important recommendations. First, if it were possible to expand the sample size for the electrophysiological recordings, that would be welcome (but not required). Next, please provide a reporting of the results of the ANOVA test, not only the results of the posthoc tests. A common format for reporting would be to provide the exact F statistic and the degrees of freedom and the p value - F(1,24) = 44.4, p<0.01. Third, please incorporate the supplemental material directly in to the main text. Fourth and finally, please also comment on the limitations inherent in using only male mice for this research. Rev1 recommends appending “in male mice” to the title. Such a revision would certainly clarify that the findings in the study might not generalize to all mice. The discussion could also discuss limitations flowing from the use of only male mice, which has the potential to place this study in context of other knowledge (or lack thereof) regarding sex as a biological variable in muscle pain.

Reviewer 1

The authors have taken an interesting clinical observation around hyaluronic acid (HA) and created a model in mice to understand how HA degradation can lead to muscle pain. The findings are novel, add to our understanding of HA actions on the nociceptive system, and are clinically relevant. My view is that this is a strong paper that will be of interest to pain neuroscientists and scientists developing HA-targeting therapeutics. I have a few suggestions to improve the paper:

1) The ephys experiments seem to show a large increase in heat responses after the hyaluronidase treatment, but the N for the experiment is small, and no effect is reported. Should the number of replicates for this experiment be increased? I am concerned that this is a false negative. Moreover, my understanding is that the muscle afferents that respond to the metabolite mixture also express TRPV1, so it would be logical that the heat response would be sensitized.

2) In Figure 3, the red panels are hard to see. Could the authors make the signal white to make visualization easier. Also, there is no quantification of the macrophage infiltration. Perhaps the authors could state why this was not done in the results. It is implied that there was no signal in the untreated mouse muscle but this could be stated more clearly.

3) In the discussion, while I agree that it is somewhat surprising that there were not changes in mRNA expression for TrkA and TRPV1, this is just mRNA and it may not reflect protein expression changes. The authors might want to note that.

4) The study only used male mice. This needs some justification, and should be more prominently noted, for instance by ending the title with the words “in male mice”.

Reviewer 2

The manuscript overall is solid; it is based on a good rationale, studies appear well-designed and rigorous, and it is well-written. There are a few issues detailed below that should be addressed before publication.

• The authors state “Responses from afferents recorded at the beginning of the experiment did not differ significantly from those recorded at the end” (Methods, p. 8 lines 179-180). How was this tested?

• It is unfortunate that the ex vivo skin-nerve-spinal cord prep recordings were performed at days 1 and 3 after PEGPH20 administration (Fig. 2; Table 2-1), given that no behavioral hindpaw tissue sensitivity was observed at these timepoints (Fig. 1B). This shortcoming should be considered when discussing the findings.

• Please could the authors provide a reference for the “low” and “high” metabolite solutions used in Fig. 2.

• LysM-cre;tdTom mice are myeloid lineage cells, not necessarily macrophages, and other cell types can express LysM (Shi et al. Methods Mol Biol. 2018; 1784: 263-275). Whilst the authors do allude to this several times, LysM-tdTom+ cells observed in the muscle are frequently referred to as “macrophages” which, though likely, may not strictly be true, and may better be described using less specific terminology.

• The anatomical figures have a few issues that would benefit from some adjustment:

o Red channels in both Fig. 3 A and C are not easily visible in the figures. It would be helpful to increase brightness (equally across vehicle and treatment groups), and perhaps show an inset or additional image showing a positively stained cell.

o There is a lack of information on quantification methods. How were sections and/or fields of view randomly selected, if at all? Was quantification performed by a blinded investigator? Were stereological correction methods used? They should be.

o Quantification of Fig. 3C would aid interpretation of the figures, rather than relying on the fact that images are representative.

o Detail on thresholding etc. for Fig. 4A-B quantification is missing.

o Please include scale bars on all images.

• In Fig. 4, the lack of behavioral measures beyond running wheel activity is a shame, especially given the confound that macrophage depletion alone reduces activity. Additional measurements, such as evoked hypersensitivity (as measured in Fig. 1B) would strengthen the authors’ claim that “macrophages are necessary for an adequate recovery after hyaluronic acid (HA) disruption.” In the absence of this data, and given the effect of macrophage depletion alone, this claim is tenuous and should be toned down or removed in the abstract, significance statement and discussion.

• Fig. 4C: including data from the AP only group for voluntary wheel activity over time on the graph (rather than within Table 4-1) would aid interpretation of AP- versus PEGPH20-induced effects.

• The authors claim that AP and PEGPH20 exert additive effects on decreases in running wheel behavior (Results, line 338 and Discussion, line 438). This additive effect is not readily apparent by eye, so the quantification supporting this statement should be fully described.

• For the qRT-PCR experiments, please could you provide evidence for whether GAPDH expression differed between experimental groups, as any variation could confound interpretation of results.

• There are some readability issues on pg.22, lines 433-440; this sentence would benefit from re-wording.
